# Musical therapy attenuates neuroma pain by modifying leptin expression

**DOI:** 10.1186/s12906-022-03795-8

**Published:** 2022-12-01

**Authors:** Yingying Lv, Junzhen Wu, Yongming Xu, Shaofeng Pu, Chen Li, Dongping Du

**Affiliations:** Pain Management Center, Shanghai Jiao Tong University Affiliated Sixth People’s Hospital, Shanghai, China

**Keywords:** Neuroma pain, Musical stimulation, Leptin

## Abstract

**Background:**

Accumulating evidence reveals that music therapy appears to help patients with pain. However, there is a limited understanding of the underlying mechanisms. Several studies indicate that leptin level has a crucial relationship with acute and chronic pain. Herein, we evaluated the effects of music stimulation and the potential roles of adipokines (leptin) in pain behaviors.

**Methods:**

We used a tibial neuroma transposition (TNT) rat model to mimic neuroma pain. Adult male Sprague–Dawley rats were randomly assigned to one of the three groups (*n* = 6):group 1 (GC), TNT with white noise; group 2(GM), TNT with music; and group 3(GH), TNT. White noise and music stimulation was given once a day following surgery until the end of the study (42^nd^ day). Pain behavioral tests were carried out before surgery and on the 3^rd^, 10^th^, 14^th^, 21^st^, 28^th^, 35^th^, and 42^nd^ days after surgery. At the end of the observation period, we analyzed the histological samples of blood, spinal cord, and prefrontal cortex to investigate the role of leptin in pain behaviors modulated by white noise and sound stimulation.

**Result:**

Music therapy might improve the pain of TNT rats. Music stimulation ameliorated paw withdrawal thermal latency (PWTL) from the 3^rd^ day after the surgery while the mechanical pain was improved 21 days after the operation.Music stimulation also increased leptin expression in the spinal cord, prefrontal cortex.White noise had no obvious effect.

**Conclusion:**

Music therapy might improve the pain of TNT rats. Besides, music stimulation ameliorated TNT-induced pain behaviors and affected leptin expression.

**Supplementary Information:**

The online version contains supplementary material available at 10.1186/s12906-022-03795-8.

## Introduction

A rise in limb amputations has been attributed to an increase in the incidence of trauma, peripheral vascular/nerve disorder, and limb malignancies. Amputation is a strategy applied to save a life in emergencies. Nonetheless, intractable postamputation pain (PAP) is challenging in clinical practice [1, 2]. Many studies suggest that the level of leptin is linked to both acute and chronic pain [3–6]. In the past two decades, music therapy has been widely used in clinical treatment, particularly for individuals suffering from depression, anxiety, and other mental illnesses [7, 8]. Although music therapy is not extensively utilized in patients with pain, studies reveal that music therapy has a positive effect on different types of pain [9, 10]. By releasing various bioactive molecules known as adipokines, such as leptin and adiponectin, adipose tissue acts as a vital endocrine organ [11]. Leptin is a 16 kDa hormone primarily produced by fat cells and secreted into the bloodstream; it was identified by positional cloning of the ob gene, a gene responsible for the development of obesity in ob/ob mice [12]. In recent years, leptin has been largely studied and characterized. Leptin is mostly known for proinflammatory activities [13]. Nevertheless, whether leptin causes pain or relieves pain remains controversial. This study aims to investigate the effects of different music on neuroma pain in rats and potential underlying mechanisms mediated by adipokines (leptin).

## Materials and methods

### Experimental animals

Adult male Sprague–Dawley rats (200–250 g) were procured from the Shanghai Laboratory Animal Center at the Chinese Academy of Science. Rats were initially housed in cages within the number upper limit and had free access to food and water. The cages were 47.5 cm in length, 35.0 cm in width, and 22.0 cm in height. Rats were maintained on a 12/12 h light/dark cycle (lights on 7:30 AM–7:30 PM). Animals were placed in individual cages after surgery. This study was carried out in compliance with the ARRIVE guidelines. All experimental protocols were approved by the Animal Care and Use Committee of Shanghai Sixth People’s Hospital affiliated with Shanghai Jiao Tong University (ethics certificate number:20160226) and performed as per the National Institutes of Health guidelines for the Care and Use of Laboratory Animals.

We established animal models as follows.The animal model of tibial neuroma transposition (TNT) was adopted as described by Dorsi et al [14]. Rats were deeply anesthetized using sodium pentobarbital via intraperitoneal injection (50 mg/kg). The tibial nerve of the rat was dissociated from the proximal 8 mm of the heel bifurcate to the distal 1 mm of the foot bifurcate, then ligated at the proximity of the plantar bifurcation with a 6–0 silk suture. Through a subcutaneous tunnel, the transected nerve terminal was gently pulled to a part 2 cm superior to the lateral malleolus and fixed beneath the skin 8-10 mm superior to the lateral malleolus. In the sham-operated rats, only the tibial nerve was dissociated and kept intact. A small piece of connective tissue was ligated and passed through the subcutaneous tunnel as per the method described above.

Experiment 1: To evaluate the effect of sound stimulation on neuroma pain, rats were randomly assigned to one of the three groups (*n* = 6): Group 1 (GC), TNT with white noise; group 2(GM), TNT with music; and group 3(GH),TNT. The evulation of the neuroma pain described by Dorsi et al. was used with minor modifications [14]. Briefly, the trial comprised 10 repetitive applications of a von Frey filament (15 g for 1–2 s) to the neuroma position at intervals of 1 to 2 min. White noise and music stimulation were administered once a day after surgery until the end of the study (42^nd^ day). Pain behavioral tests were carried out before surgery and on the 3^rd^, 10^th^, 14^th^, 21^st^, 28^th^, 35^th^, and 42^nd^ days after surgery. The rats of GH were placed in the room for the same duration, yet without any sound stimuli.

Experiment 2: To investigate the role of leptin in pain behaviors modulated by white noise and sound stimulation.

The rats were sacrificed after the behavior test on the 42^nd^ day,the end of the observation period.We analyzed the histological samples of blood, spinal cord, and prefrontal cortex. Leptin levels in plasma were assayed using Rat Leptin ELISA kit (Crystal Chem Inc., Chicago, IL, USA) following the manufacturer’s protocols.The concentration of leptin in the spinal cord and prefrontal cortex was measured by Western blotting.

### Music stimuli

The acoustic stimuli towards the subjects of the different groups were conducted in a soundproofed room with the ambient sound pressure of 40 dB SPL. The animals were unrestrained in a cage 60 cm below the loudspeaker for the acoustic stimuli. The GM group TNT rats were exposed to a piece of traditional Chinese music “LiangZhu” played by the Erhu with the intensity between 50 dB SPL and 55 dB SPL monitored by a ¼-inch microphone linked to a sound level meter (Microphone: 2520, SLM: 824, Larson Davis, Depew, NY, USA). The GC was exposed to the white noise with the intensity of 52 dB SPL. The animals of these two groups were exposed for 30 min per day and the total duration of the acoustic exposure was 42 days. The rats of GH were placed in the room for the same duration, yet without any sound stimuli.

### Evaluation of pain behaviors

1. Von Frey's up-down method. The behavioral tests were performed in a quiet and appropriate room by an experienced observer blinded to the different treatments. The rats were allowed to adjust to the testing environment for 20 min before testing. The evaluation of the neuroma pain approach described by Dorsi et al [14] was used with minor modifications. The trial comprised 10 repetitive applications of a von Frey filament (15 g for 1–2 s) to the neuroma position at intervals of 1 to 2 min. The paw withdrawal threshold was measured using the von Frey up-down method described by Chaplan et al [15]. The test began with a middle filament in the series (2.0 g). An ascending series of von Frey filaments of logarithmically incremental force (0.6, 1.0, 1.4, 2.0, 4.0, 6.0, 8.0, 10.0, and 15.0 g; Stoelting) were applied to the lateral plantar surface of the hind paw. A positive response was a rapid withdrawal and/or licking of the paw immediately after stimulus application. Whenever a positive or negative response occurred, the testing proceeded for five more stimuli after the first change in response, and the pattern of responses was converted to a 50% paw withdrawal threshold (50% PWT) as described by Sun et al. [6].
2. Paw withdrawal thermal latency (PWTL). According to the Hargreaves method, the rats were placed in plexiglass chambers, and a radiant beam of light was applied to the hind paw through a glass floor [16]. Paw withdrawal latencies were recorded in duplicate per paw. A third measurement would be taken if the second latency recorded was not within 64 s of the first. The values of the two closest latencies were averaged to establish the overall latency to withdrawal.

### Histology analysis

Rats were deeply anesthetized using an intraperitoneal injection of 100 mg/kg sodium pentobarbital and blood was collected by cardiac puncture. Subsequently, the spinal cord and prefrontal cortex of all rats were harvested and immediately stored at -80℃, to be processed for use in real-time quantitative PCR or Western blotting. The same was done with blood samples.

1. Enzyme-linked immunosorbent assay. After the pain behavior test, blood was collected 42 days after the operation. The plasma was separated by centrifugation at 5000 g for 5 min and the supernatants were obtained for subsequent protein analysis. Rat Leptin ELISA kit (Crystal Chem Inc., Chicago, IL, USA) was used to analyze the leptin levels in plasma.
2. Western blotting. In tissue protein extraction buffer (Thermo Fisher), the obtained spinal cord or prefrontal cortex was homogenized with a protease inhibitor cocktail (Roche). The BCA Protein Assay Kit was used to establish the protein concentrations of samples. Thereafter, the proteins were separated using the SDS-PAGE, then transferred to PVDF membranes (Merck Millipore). The membranes were cut prior to hybridisation with antibodies by the location of the target protein according to the pre staining results.After blocking with 5% bovine serum albumin at room temperature for 1 h, the membranes were incubated overnight at 4℃ with the following primary antibodies: anti-leptin(1:1000, rabbit polyclonal IgG; Invitrogen) and anti-β actin (1:1000, mouse monoclonal IgG; Santa Cruz). Afterward, the membranes were incubated with HRP-conjugated secondary antibodies (1:5000; HuaAn Biotechnology) at room temperature for 1 h. The membranes were rinsed using Tris-buffered saline and 0.1% Tween 20 between the above steps. Eventually, blots were detected via enhanced chemiluminescence substrate solution (ThermoFisher). In the chemiluminescent instrument darkroom, the blotting membranes were exposed to the X-ray Film,then printed out, scanned (Imacon 949) and quantified using the ImageJ software (version 2.0.0, National Institutes of Health, Rockville, MA, USA).

### Statistical analysis

All datas were expressed as means ± SEM.Statistical analyses were performed using statistical packages (GraphPad Prism 5, GraphPad, San Diego, CA). The Student’s t-test was used to compare the difference between two groups, whereas a two-way ANOVA test was used to compare the difference among three groups. A *P* value of less than 0.05 (*P* < 0.05) was considered statistically significant.

## Results

### Music stimulation ameliorates TNT-induced pain behaviors

**Fig. 1:**
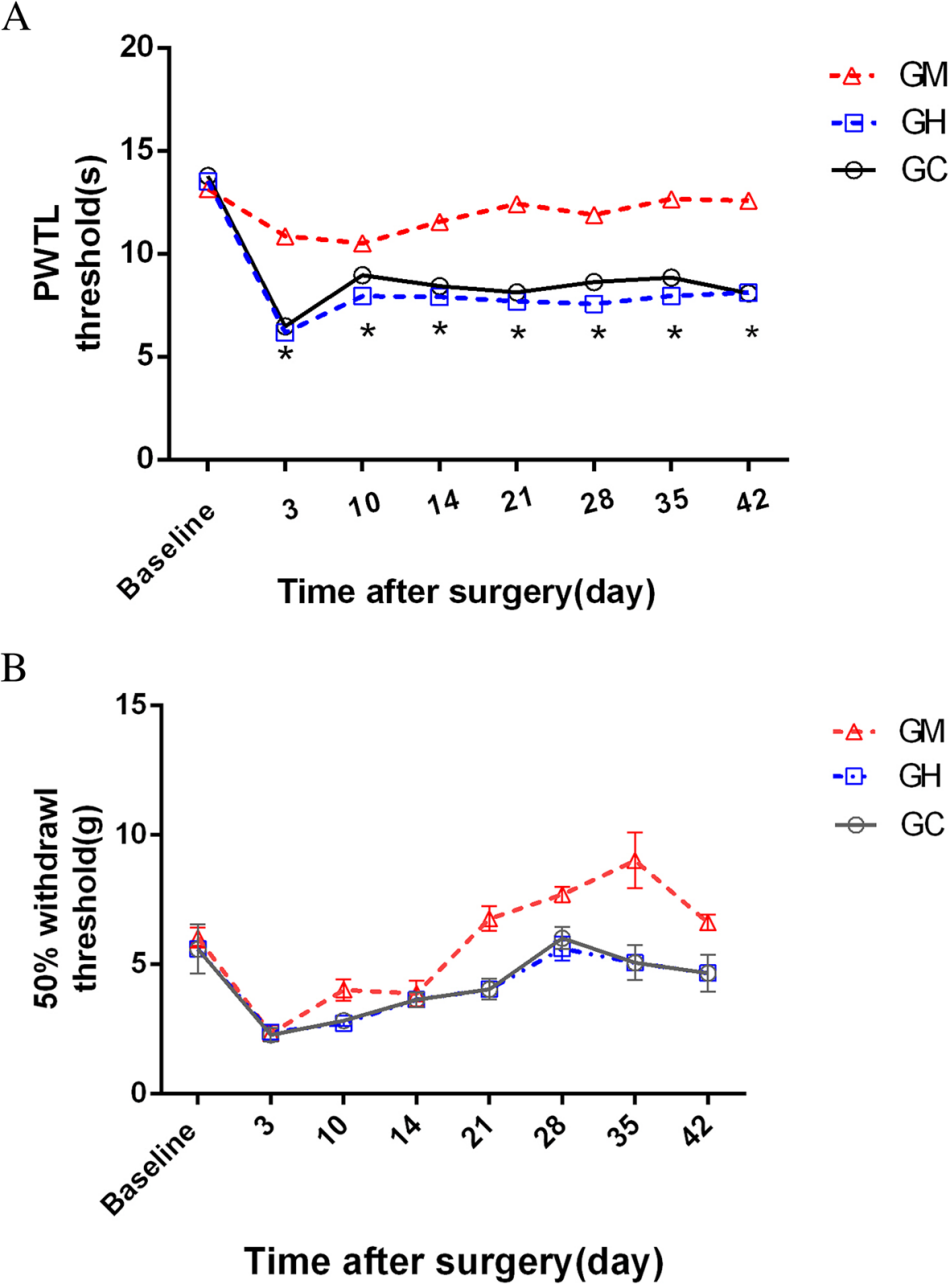
**A** Effects of music stimulation on paw withdrawal latency to thermal heat induced by TNT surgery. No significant difference was observed between the three groups at baseline (*P* > 0.05). TNT surgery reduced the response time of paw withdrawal latency to thermal heat on the neuroma site at all test time points after surgery in GC and GH. A slight change was noted in GM. (*P < 0.05). GM compared with GC and GH, All rats assigned to different groups  6 for each group. B. Effects of music stimulation on behavioral pain induced by TNT surgery. TNT surgery decreased the 50% paw withdrawal threshold in response to mechanical stimuli to the hind paw plantar after surgery, no significant difference was noted among the three groups during the first 14 days after the operation (*P* > 0.05). Music stimulation reversed it from 21^st^ day to the 42.^nd^ day (*P < 0.05)

All rats assigned to different groups exhibited similar baselines of pain behaviors before surgery (Fig. 1A, B; *P* > 0.05). After the operation, All the TNT rats developed increased responsiveness to mechanical and thermal stimuli at the neuroma area compared to the baselines on the 3^rd^ day after the operation (*P* < 0.01). After receiving music stimuli three days after the surgery, PWLT of GM group TNT rats significantly increased compared to that in the other two groups (F = 43.24, *P* < 0.01). The effect lasted to the 42^nd^ day. But the significant difference of mechanical pain presented from the 21^st^ day (GC and GH vs GM:8.12 ± 8.23 and 7.70 ± 0.22 vs 12.42 ± 0.26,*P* < 0.01) to the 42^nd^ day(GC and GH vs GM:8.08 ± 1.10 and 8.10 ± 0.25 vs 12.42 ± 0.27,*P* < 0.01). There was no statistical difference among the three groups of 50% PWT until the 21^st^ day (GC and GH vs GM:4.04 ± 0.33 and 4.04 ± 0.33 vs 6.77 ± 0.39,*P* < 0.01). The effect also lasted to the 42^nd^ day. At the end of the experiment, we noted that music stimuli ameliorated PWLT from the 3^rd^ day and the mechanical pain improved 21 days after the operation in GM group rats. Both effects lasted until the 42nd day (Fig. 1A, B). At the same time, white noise demonstrated no remarkable effect.

### More leptin expression was associated with improved pain behaviors in the music stimulation groups

**Fig. 2:**
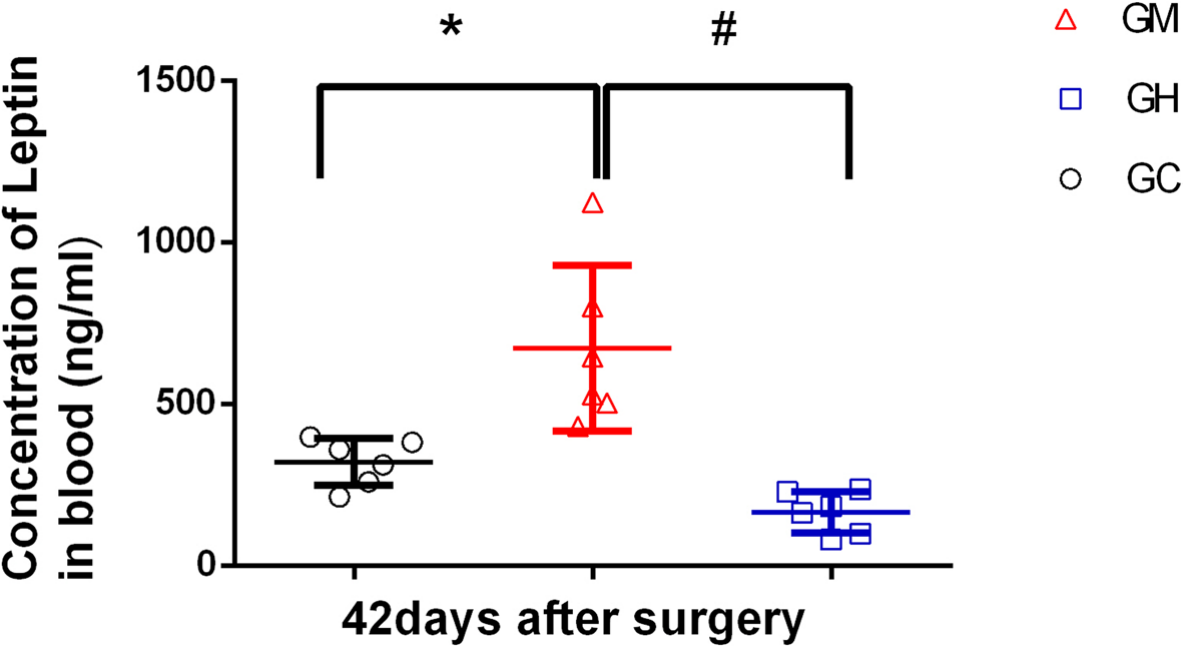
Leptin level in plasma after 42 days in each group. GM concentration was significantly higher than that of the other two groups. A significant difference in the Leptin level was evident between GM and GH, GM and GC (*,#*P* < 0.01)

**Fig. 3:**
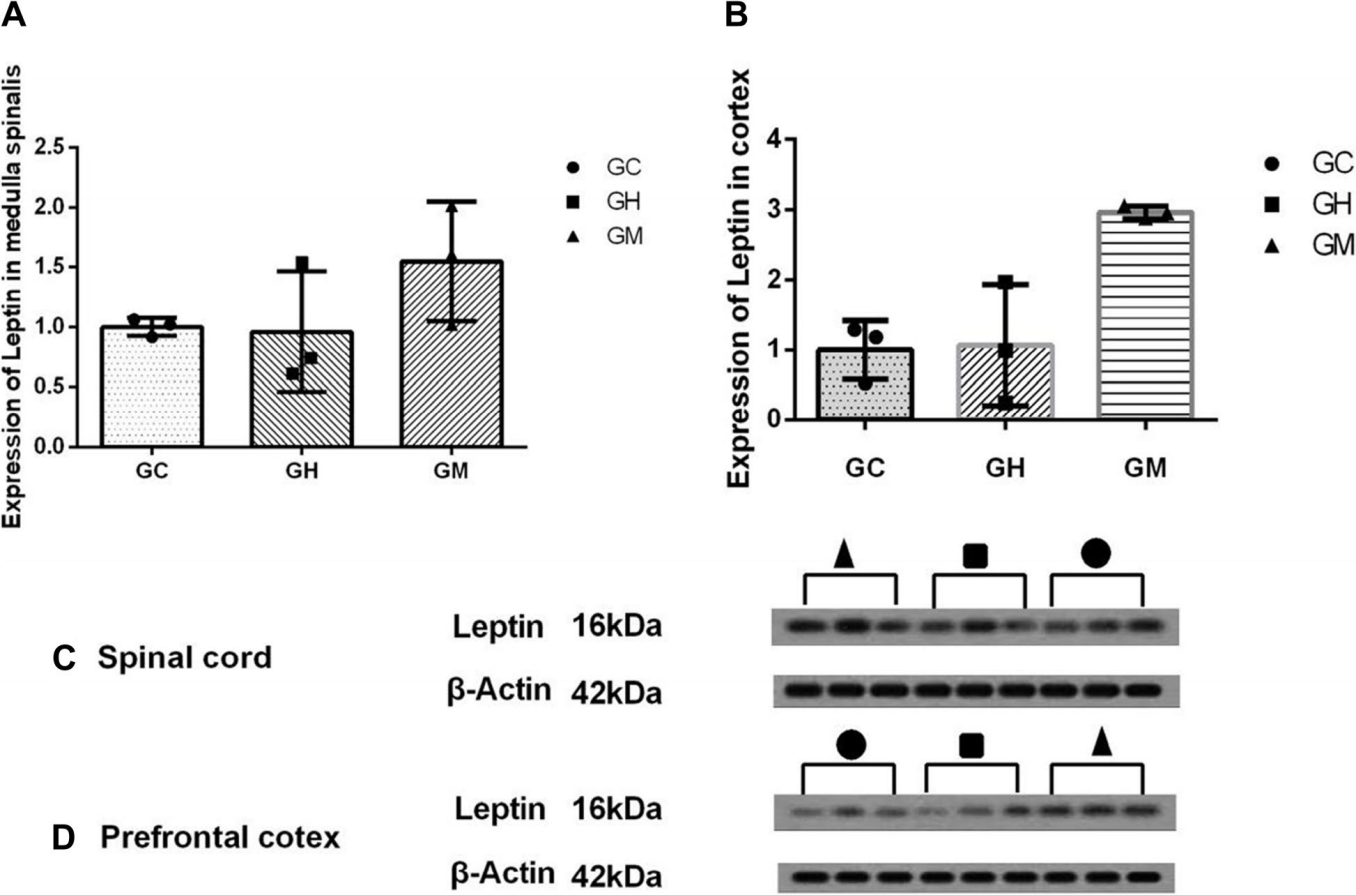
Effects of music stimulation on expression of leptin receptor in the spinal cord and prefrontal cortex on 42.^nd^ day. **A** and **B** Western blotting showing a significantly upregulated expression of leptin in the GM group in the prefrontal cortex. No significant difference was noted in the spinal cord. **C** and **D** Westen blot of the leptin in spinal cord and cortex of TNT rats. The membranes were cut prior to hybridisation with antibodies.We confirmed that music stimulation influences the leptin changes. In conclusion, no significant difference was noted in leptin expression among the three groups in the spinal cord (Fig. 3A *P* > 0.05). The difference between GM and the other two groups was statistically significant in prefrontal cortex (Fig. 3B*P < 0.01)

On the 42^nd^ day after the behavior test, leptin expression in peripheral blood among the three groups were significantly different (Fig. 2, F = 21.25,*P* < 0.01). Leptin expression in plasma in the GM group was significantly higher than that in the other two groups (Fig. 2,GC and GH vs GM:320.60 ± 72.69 and 164.70 ± 64.40 vs 672.10 ± 256.90,*,#*P* < 0.01). Meanwhile, leptin expression in the the prefrontal cortex increased in GM, and reduced in GH. This was confirmed by the findings of Western blot. Significant differences were observed among GM, GC, and GH (Fig. 3A,B,C&D,*P < 0.01), however, no significant difference was found between GC and GH (*P* > 0.05). Besides, we found no significant difference in leptin expression among the three groups in the spinal cord.

## Discussion

The most representative type of chronic pain is neuropathic pain. It causes long-term spontaneous pain and produces various accompanying emotional disorders (including anxiety, depression, etc.) and cognitive impairment. In our experiment, we found music therapy relieves neuroma pain. White noise had no significant effect. Over the last 4 decades, research on the effects of music on the brain has remarkably advanced [17]. For instance, Wiśniewska discovered that music therapy can relax old horses and relieve pain caused by degeneration [10]. Despite numerous clinical reports on the benefits of music in pain management under different conditions more than 10 years ago [9, 18, 19], the underlying neuronal mechanisms remain unknown. Music therapy is globally used in clinical treatment, specifically in patients with depression, anxiety, and other psychological disorders. It influences the sympathetic and parasympathetic systems by the heart rate. Besides, it is responsible for the emotional excitability of the body [20, 21]. Notably, we demonstrated smooth music can increase pain threshold and leptin expression in TNT mice. The crucial aspect of music is based on sound vibration, i.e., due to its deep penetration into the brain and body, it positively or negatively affects the mood and emotions at both the behavioral and neuronal levels [22]. In recent years, the impact of leptin on pain has been a hot topic of research. Lim et al. reported that leptin related nociceptive behavior was due to its promotion of nerve injury, thereby indicating a cellular relationship between the spinal effect and the cellular mechanism mediated by NMDA receptor of neuropathic pain [23, 24]. Many studies, though mostly in vitro, confirmed leptin’s “bad reputation”. However, some studies pointed out that leptin has a positive effect on cartilage repair and pain relief. Fu R et al. reported that leptin advanced cell proliferation and chondrocyte genetic expression in a dose-dependent manner [25]. Li et al. found that lack of leptin receptor gene may cause loss of bone mass in the vertebra [26]. This may be related to pain caused by osteoarthritis and intervertebral disc degeneration. Leptin is primarily secreted by adipose tissue. Under normal circumstances, adipose tissue rarely produces extra leptin; however, it raises in some pathological states such as inflammation [27]. Paiva ES et al. found that patients with fibromyalgia and overweight /obesity had lower leptin levels, compared with the controls [28]. Some researches suggested leptin plays a significant role in the regulation of energy balance, neuroendocrine system, immune function,which is achieved by affecting the central nervous system and peripheral tissues [29, 30]. Leptin receptors are highly expressed in areas including hypothalamic cortex, hippocampus and amygdala [31, 32]. In some brain studies, leptin plays an antidepressant role in stress models, which may be related to its function of regulating dopaminergic neurotransmission [33, 34]. The experiments of peripheral and intrahippocampal injection proved that leptin has direct antidepressant properties [35, 36]. In the researches of neurological diseases such as Parkinson's disease, Alzheimer's disease and multiple sclerosis, it is found that leptin shows neuroprotective properties [37–39]. Some studies showed this neuroprotective effects in Parkinson's disease have been determined through in vivo and in vitro studies [37, 40].

In our study, leptin expression increased in the prefrontal cortex, and blood in the GM group rats. Among the three groups, we found no difference in leptin expression at the medulla spinalis. We selected the prefrontal cortex (PFC) as the target for studying leptin concentration. The findings displayed a positive expression in GM compared to that of the other two groups. The prefrontal cortex is crucial in pain processing, which is dependent on its connections to other areas of the cerebral neocortex, hippocampus, periaqueductal gray, thalamus, amygdala, and basal nuclei. It changes during acute and chronic pain [41]. Music regulates pain responses in different parts of the CNS. In addition to animal research, this field is of significance in clinical research. When listening to pleasurable music, Dobek found increased activity in brain regions i.e., parts of descending pain modulatory system [42]. Additional studies indicate that PFC changes can be reversed after a successful pain control [43, 44].

Neuropathic pain and its complications induce extreme pain in patients and severely influence the quality of life. Music intervention is a type of non-drug therapy with a long history. It is clinically utilized as an auxiliary therapy for postoperative recovery and relieving postoperative pain. Researchers believe that this may influence the sympathetic and parasympathetic nervous systems via sound vibration [20, 45, 46]. In addition,mental disorder can influence the experience of pain, impacting its perception, affective response and the interpretation of physical symptoms.Liu et al. suggested that pain and depression tend to occur simultaneously and share some common neural circuits and neurotransmitters [47]. Angioli R et al. demonstrated that anxiety state is highly related to pain perception, music therapy can relieve anxiety so as to reduce pain [48]. All in all, pain is not an emotionally pure experience, but always accompanied by emotional suffering and depression. We consider that music therapy may prevent stress depression in rats with nerve injury, and stimulate the brain tissue to secrete leptin, which played a neuroprotective role, so as to reduce their pain sensitivity,which would be our follow-up experiments.

## Conclusion

In contrast with behavioral pain outcomes, our findings suggest that the higher the leptin level in tissues, including peripheral blood and cerebral cortex, the higher the mechanical and thermal pain thresholds.

According to the findings obtained in the neuroma pain model, we conclude that graceful music influence leptin concentration both in the peripheral and central nervous systems. Our work revealed that soothing music can improve neuroma pain in TNT rats via the leptin pathway. Moreover, we provide a mechanism that may yield beneficial effects of music on pain conditions. Adipokines are potential targets for the treatment of neuropathic pain, this should be confirmed by additional research.

## Supplementary Information

**Additional file 1.**

## Data Availability

All data generated or analysed during this study are included in this published article.
