# Supplementary material for: Musical therapy attenuates neuroma pain by modifying leptin expression
Source: BMC Complement Med Ther. 2022 Dec 1;22:316. doi: 10.1186/s12906-022-03795-8 (PMC9714090; doi:10.1186/s12906-022-03795-8)
Supplement: Supplementary file 1 — Additional file 1. [file 12906_2022_3795_MOESM1_ESM.docx]

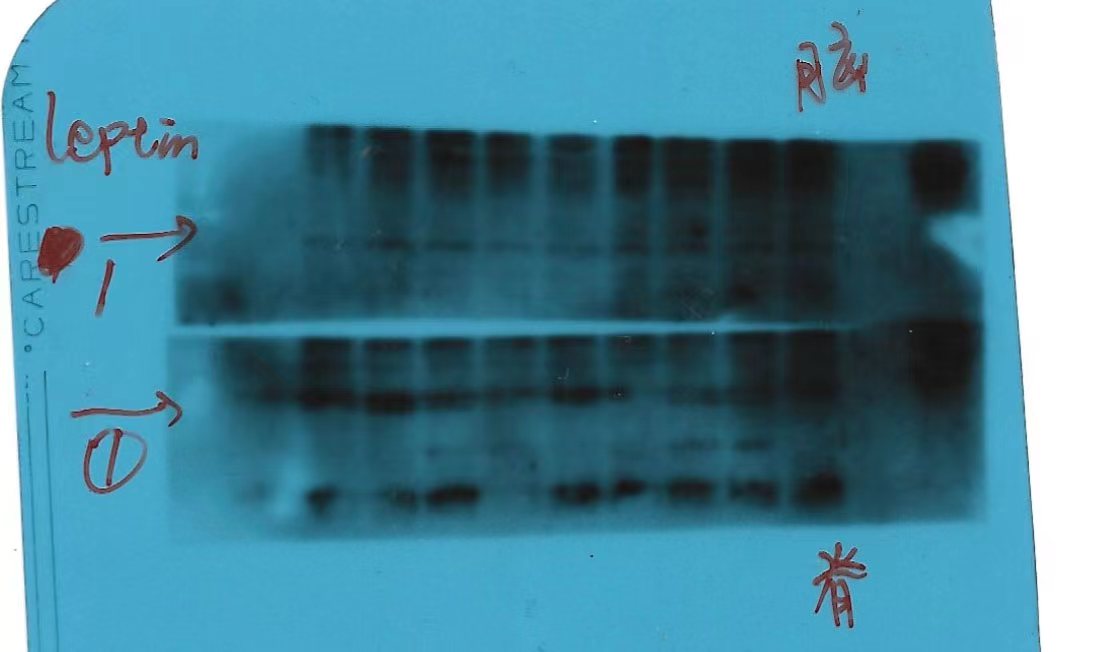


X-ray film:the first line was the expression of leptin in brain,the second line was the expression of leptin（16kDa） in spinal cord.


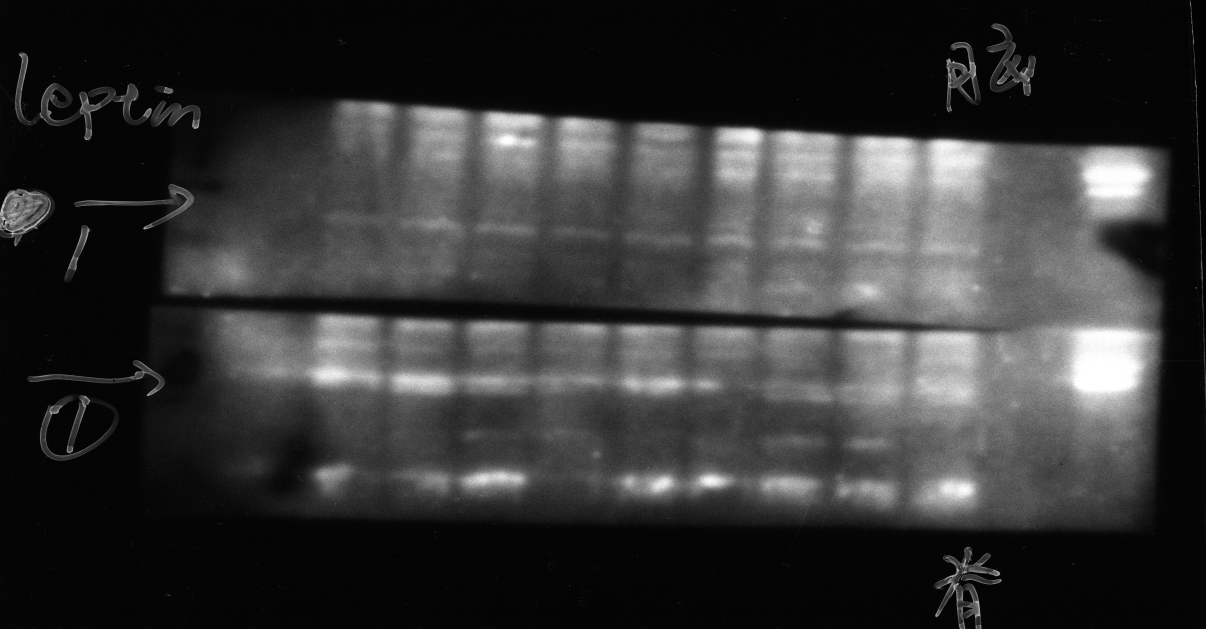


High-resolution X-ray film scan to check the bands.


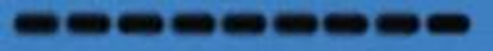


Original exposure image of β-Actin（42kDa） for spinal cord


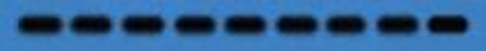


Original exposure image of β-Actin（42kDa） for prefrontal cortex
